# Interleukin-22 ameliorated renal injury and fibrosis in diabetic nephropathy through inhibition of NLRP3 inflammasome activation

**DOI:** 10.1038/cddis.2017.292

**Published:** 2017-07-20

**Authors:** Shaofei Wang, Yubin Li, Jiajun Fan, Xuyao Zhang, Jingyun Luan, Qi Bian, Tao Ding, Yichen Wang, Ziyu Wang, Ping Song, Daxiang Cui, Xiaobin Mei, Dianwen Ju

**Affiliations:** 1 Department of Microbiological and Biochemical Pharmacy, Key Lab of Smart Drug Delivery, Ministry of Education, School of Pharmacy, Fudan University, Shanghai 201203, China; 2 Department of Nephrology, Changhai Hospital, Second Military Medical University, Shanghai 200433, China; 3 Collaborative Innovation Center of Systems Biomedicine, Shanghai Jiao Tong University, School of Medicine, Shanghai 200240,China

## Abstract

Diabetic nephropathy (DN) is one of the most lethal complications of diabetes mellitus with metabolic disorders and chronic inflammation. Although the cytokine IL-22 was initially implicated in the pathogenesis of chronic inflammatory diseases, recent studies suggested that IL-22 could suppress inflammatory responses and alleviate tissue injury. Herein, we examined the role of IL-22 in DN. We found that serum levels of IL-22 were significantly downregulated in both patients and mice with DN. The expression of IL-22 was further decreased with the progression of DN, whereas IL-22 gene therapy significantly ameliorated renal injury and mesangial matrix expansion in mice with established nephropathy. IL-22 could also markedly reduce high glucose-induced and TGF-*β*1-induced overexpression of fibronectin and collagen IV in mouse renal glomerular mesangial cells in a dose-dependent manner, suggesting the potential role of IL-22 to inhibit the overproduction of ECM *in vitro*. Simultaneously, IL-22 gene therapy drastically alleviated renal fibrosis and proteinuria excretion in DN. In addition, IL-22 gene therapy markedly attenuated hyperglycemia and metabolic disorders in streptozotocin-induced experimental diabetic mice. Notably, IL-22 drastically reversed renal activation of NLRP3, cleavage of caspase-1, and the maturation of IL-1*β* in DN, suggesting unexpected anti-inflammatory function of IL-22 via suppressing the activation of NLRP3 inflammasome *in vivo*. Moreover, IL-22 markedly downregulated high glucose-induced activation of NLRP3 inflammasome in renal mesangial cells in a dose-dependent manner, indicating that the effects of IL-22 on NLRP3 inflammasome activation was independent of improved glycemic control. These results suggested that nephroprotection by IL-22 in DN was most likely associated with reduced activation of NLRP3 inflammasome. In conclusion, our finding demonstrated that IL-22 could exert favorable effects on DN via simultaneously alleviating systemic metabolic syndrome and downregulating renal NLRP3/caspase-1/IL-1*β* pathway, suggesting that IL-22 might have therapeutic potential for the treatment of DN.

Diabetic nephropathy (DN), characterized clinically by progressive increase in proteinuria, and pathologically by excessive deposition of extracellular matrix (ECM) components, and subsequent glomerulosclerosis and tubulointerstitial fibrosis, is the leading cause of end-stage renal disease (ESRD) worldwide. It has been generally recognized that the development and progression of DN are attributed to multiple interconnected mechanisms, including initial systemic pathogenesis such as hyperglycemia and metabolic disorders, as well as subsequent renal pathogenesis such as fibrosis and inflammation.^1, 2, 3, 4^ Thus, it is crucial to identify a novel therapy for DN that simultaneously improves the systemic pathogenesis and renal pathogenesis to protect against diabetic kidney complications.^5, 6, 7, 8^

IL-22, a member of IL-10 cytokine superfamily, is dominantly produced by innate lymphoid cells and CD4^+^ T helper subtypes such as Th17 and Th22 cells.^9, 10^ IL-22 could play either a protective or pathogenic role in the onset and progression of inflammatory and autoimmune diseases.^11, 12, 13, 14, 15, 16, 17^ It has been indicated that IL-22 pathway is indispensable for maintaining metabolic homeostasis, and administration of IL-22 alleviates metabolic disorders including hyperglycemia and dyslipidemia.^18, 19, 20^ Furthermore, recent studies have reported that IL-22 effectively ameliorates renal injury and preserves renal function in acute kidney injury by suppressing inflammation.^21, 22^ Although these observations implicate the importance of IL-22 in metabolism modulation and renal protection, the role of IL-22 in the progression of DN has not been defined, nor has its underlying molecular mechanism been determined. Activation of NOD-like receptor family pyrin domain-containing protein 3 (NLRP3) inflammasome is an important contributor to renal inflammation and fibrosis in chronic kidney disease via processing and secretion of the pro-inflammatory cytokines IL-1*β* and IL-18.^23, 24, 25, 26, 27, 28^ Notably, it has been elucidated by late-breaking studies that activation of NLRP3 inflammasome participates in the onset and progression of DN and targeting NLRP3 inflammasome activation may be a feasible therapeutic approach for DN.^29, 30, 31, 32^ However, it remains unknown whether IL-22 could exert beneficial effects on DN via modulation of NLRP3 inflammasome activation or not. Streptozotocin-induced mouse model of experimental DN is widely recognized animal model of diabetic kidney injury with similarities to human diabetic kidney disease (DKD). Although C57BL/6 mice have been reported to be somewhat genetically resistant to the development of DN, they are susceptible to experimental DN induced by multiple low-dose STZ injections that has been considered a reliable protocol to obtain sufficient diabetes to cause renal injury.^33, 34, 35^ Thus, we employed the commonly used C57BL/6 mice to establish mouse model of DN.

In the present study, we aimed to examine the role of IL-22 in DN and determine whether IL-22 could exert comprehensive therapeutic effects via both systemic and local mechanisms. Investigating the therapeutic effects and deciphering the underlying mechanisms of IL-22 could lead to the development of novel therapies for DN, thus providing scientific basis for therapeutic strategies for DKD on the basis of simultaneous regulation of metabolism and NLRP3 inflammasome activation.

## Results

### IL-22 expression was abnormally decreased in patients and mice with DN

**Figure 1:**
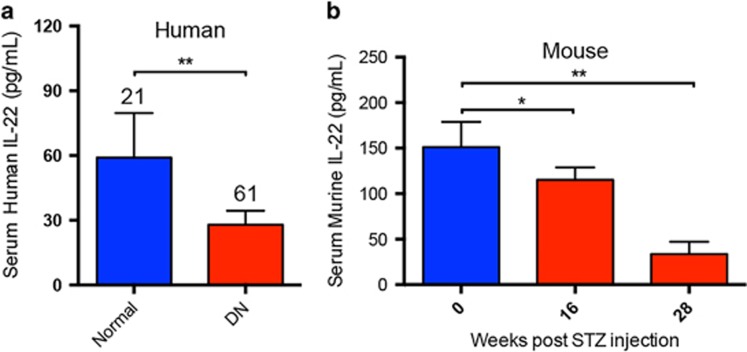
Downregulation of serum IL-22 in patients and mice with diabetic nephropathy. (**a**) Serum levels of IL-22 in patients with DN and age-matched healthy controls measured by ELISA assay. Numbers of human serum samples analyzed in each group were indicated at the top of each bar. (**b**) Serum levels of IL-22 in mice with established DN and age-matched normal controls measured by ELISA assay (*N*=5). *P<0.05; **P<0.01

To explore the possible association between the expression of IL-22 and the development of DN, we quantified the serum levels of IL-22 in patients and mice with DN, respectively. As shown in Figure 1a, IL-22 expression was significantly decreased in patients with DN as compared with age-matched healthy controls. Correlation analysis between IL-22 expression and disease duration showed that serum levels of IL-22 in patients with DN were negatively associated with the progression of DKD (*r*=−0.284; *P*=0.027). Meanwhile, serum levels of IL-22 were also downregulated in mice with established DN. Moreover, IL-22 expression was further reduced with the development of DN (Figure 1b), further suggesting that downregulation of IL-22 may correlate with the progression of DKD.

### IL-22 gene therapy attenuated renal injury and mesangial matrix expansion in established nephropathy

To determine the role of IL-22 in DN, we constructed eukaryotic expression plasmid DNA encoding murine IL-22 (Supplementary Figure 1A). The efficacy of gene delivery via the recombinant plasmid to cells was determined by immunoblot analysis. As expected, the intracellular expression of IL-22 in HEK293T cells transiently transfected with pVAX1mIL22 was substantially increased as compared to that of cells transfected with empty vector (Supplementary Figures 1B and C). Meanwhile, to determine the effectiveness of pVAX1 plasmid vector-mediated gene transfer of murine IL-22 *in* *vivo*, time-dependent variation of serum IL-22 following intramuscular injection of the recombinant plasmid was analyzed by ELISA assay (Supplementary Figure 1D). As shown evidently in Supplementary Figure 1E, a pronounced increase of serum levels of murine IL-22 was observed in mice after intramuscular gene transfer of IL-22, which peaked on day 3 after injection and almost completely subsided by day 10. On the basis of kinetics study, therapeutic plasmid was administered at weekly intervals in the subsequent therapeutic intervention to maintain the high expression of the transgene.

**Figure 2:**
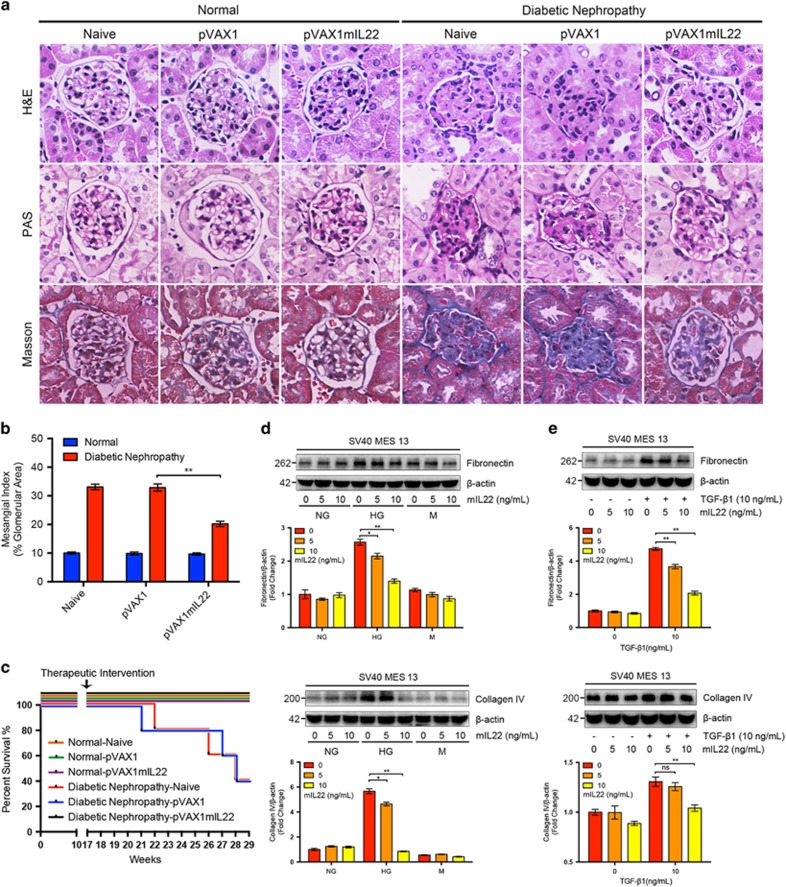
Attenuated renal injury and mesangial matrix expansion with established diabetic nephropathy after IL-22 gene therapy. (**a**) Representative micrographs of H&E staining, PAS staining, and Masson staining. Original magnification: × 400. (**b**) Glomerular mesangial matrix expansion quantified from PAS staining. (**c**) Survival rate of mice after IL-22 gene therapy. (**d**–**e**) Reduced synthesis of high glucose-induced and TGF-*β*1-induced ECM proteins in renal glomerular mesangial cells after IL-22 treatment. Representative immunoblots and semi-quantitative analysis of cytosolic expression of fibronectin and collagen IV from three independent experiments was carried out using ImageJ (National Institutes of Health, Bethesda, MD, USA). Densitometric values of immunoreactive bands were normalized to those of *β*-actin and the results were expressed as fold changes. *P<0.05; **P<0.01; NS, no significance

Next, we employed the long-term mouse model of STZ-induced experimental DN to assess the therapeutic effects of IL-22 gene therapy on DKD. IL-22 gene therapy was started at 17 weeks after the last STZ injection and continued for 12 weeks to determine the therapeutic effects of IL-22 on the progression of DKD. As it was clearly elucidated in Supplementary Figures 2 and 3, IL-22 gene therapy was initiated in mice with established nephropathy and continued for 12 weeks to determine the therapeutic effects of IL-22 on the progression of DKD. After administration of therapeutic plasmid pVAX1mIL22 via direct intramuscular injection for 12 consecutive weeks, renal pathological alterations, glycogen deposition, and collagen accumulation were substantially improved in mice with established nephropathy as indicated by hematoxylin and eosin (H&E) staining, periodic acid–Schiff (PAS) staining, and Masson staining, respectively (Figure 2a). Notably, apparent glomerular mesangial matrix expansion was observed in diabetic mice, which was obviously reduced after IL-22 gene therapy (Figure 2b). In addition, IL-22 gene therapy could prevent death of mice with established nephropathy (Figure 2c). Interestingly, IL-22 markedly reduced high glucose-induced and TGF-*β*1-induced overexpression of ECM proteins such as fibronectin and collagen IV in mouse renal glomerular mesangial cells and human renal tubular epithelial cells in a dose-dependent manner, suggesting the potential role of IL-22 to inhibit the overproduction of ECM *in vitro* (Figures 2d, e, Supplementary Figures 4A and B).

### IL-22 gene therapy alleviated renal fibrosis in mice with established nephropathy

**Figure 3:**
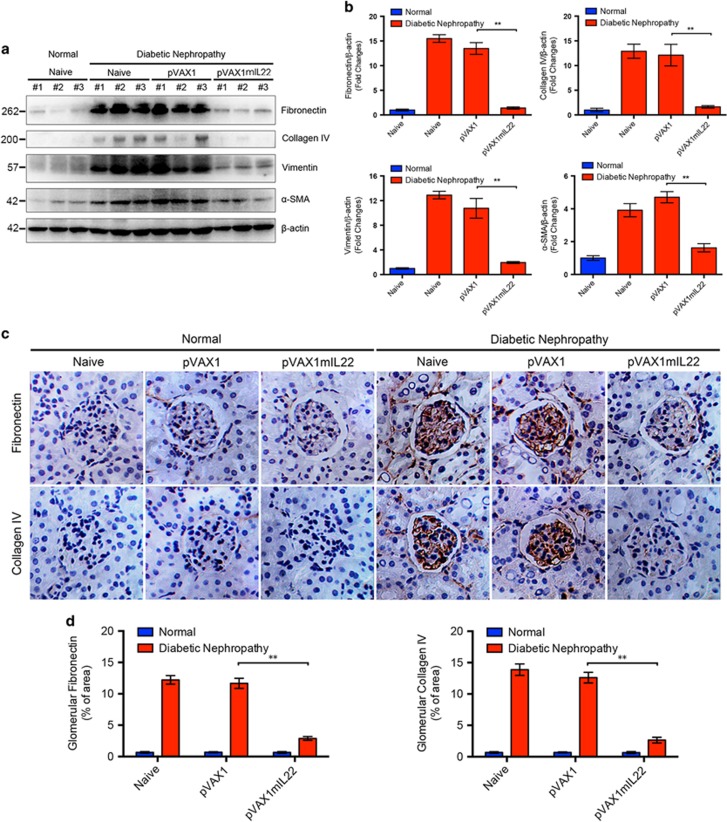
Alleviated renal fibrosis in mice with established nephropathy after IL-22 gene therapy. (**a**) Renal expression of fibronectin, collagen IV, vimentin, and *α*-SMA by immunoblot analysis. (**b**) Quantitative analysis of renal expression of fibronectin, collagen IV, vimentin, and *α*-SMA from three individual experiments. (**c**) Deposition of the ECM proteins including fibronectin and collagen IV in glomeruli by immunohistochemistry analysis. Original magnification: × 400. (**d**) Qualification of glomerular fibronectin and collagen IV expression by ImageJ. Densitometric values of immunoreactive bands were normalized to those of *β*-actin and the results were expressed as fold changes. **P<0.01

Renal fibrosis, characterized by excessive accumulation of ECM, plays a central role in the development of DN. Thus, we next examined whether the blockage of diabetic kidney injury by IL-22 gene therapy was mediated by inhibition of renal fibrosis. As shown in Figure 3a, IL-22 suppressed diabetes-induced glomerular and interstitial fibrosis as indicated by reduced fibronectin, collagen IV, vimentin and *α*-SMA expression in kidney tissue lysates. In parallel, semi-quantitative immunoblot analysis also showed significant decreased protein expression of fibronectin, collagen IV, vimentin, and *α*-SMA in mice with established nephropathy after IL-22 gene therapy (Figure 3b). Immunohistochemical staining of collagen IV and fibronectin was performed to further clarify the effect of IL-22 on renal fibrosis (Figure 3c). Although increased fibronectin and collagen IV were detected in tubulointerstitial compartment, fibronectin and collagen IV were predominately accumulated in the mesangial area of glomeruli in diabetic mice, demonstrating the occurrence of diffuse mesangial expansion and sclerosis. Conversely, both proteins were expressed at lower levels in the glomeruli of diabetic mice treated with pVAX1mIL22, compared with the naive group and vector-treated group (Figure 3d). In brief, our data indicated that renal fibrosis, especially glomerular fibrosis, was significantly attenuated after IL-22 gene therapy in established DN.

### IL-22 gene therapy preserved renal function in experimental DN

**Figure 4:**
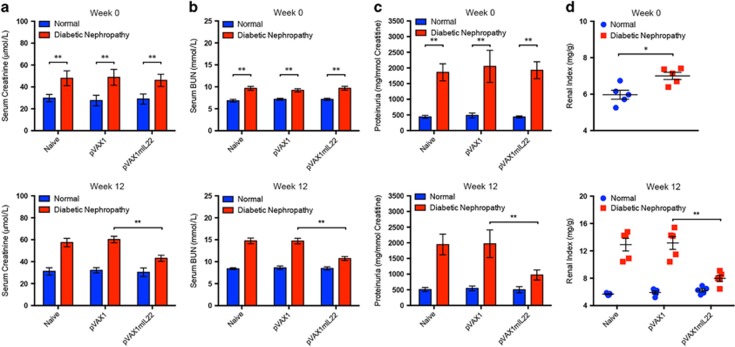
Improved renal function in mice with experimental diabetic nephropathy after IL-22 gene therapy. (**a**) Serum creatinine levels, (**b**) serum BUN levels, (**c**) proteinuria and (**d**) renal index of mice before and after IL-22 gene therapy for 12 weeks. Renal index=renal weight (mg)/body weight (g). *N*=5; *P<0.05; **P<0.01. The time point of the initiation of IL-22 gene therapy was defined as week 0

Serum creatinine and BUN, generally considered as makers of renal function, were determined to evaluate the effect of IL-22 on renal dysfunctions in mice with established experimental DN. Consistent with histopathological analysis, serum levels of creatinine and BUN of diabetic mice in naive group and vector control group were significantly elevated. In contrast, administration of therapeutic plasmid pVAX1mIL22 effectively downregulated serum levels of creatinine and BUN, implying largely improved renal dysfunctions after IL-22 gene therapy (Figures 4a and b). Moreover, the development of proteinuria, a clinical predictor of renal lesions in DN, was also obviously attenuated by IL-22 gene therapy (Figure 4c). In addition, IL-22 gene transfer led to significant reduction of diabetes-induced increase of kidney index (Figure 4d). Collectively, these data suggested that IL-22 gene therapy effectively restored renal function in experimental DN.

### IL-22 gene therapy alleviated hyperglycemia and improved metabolic disorders of diabetic mice

**Figure 5:**
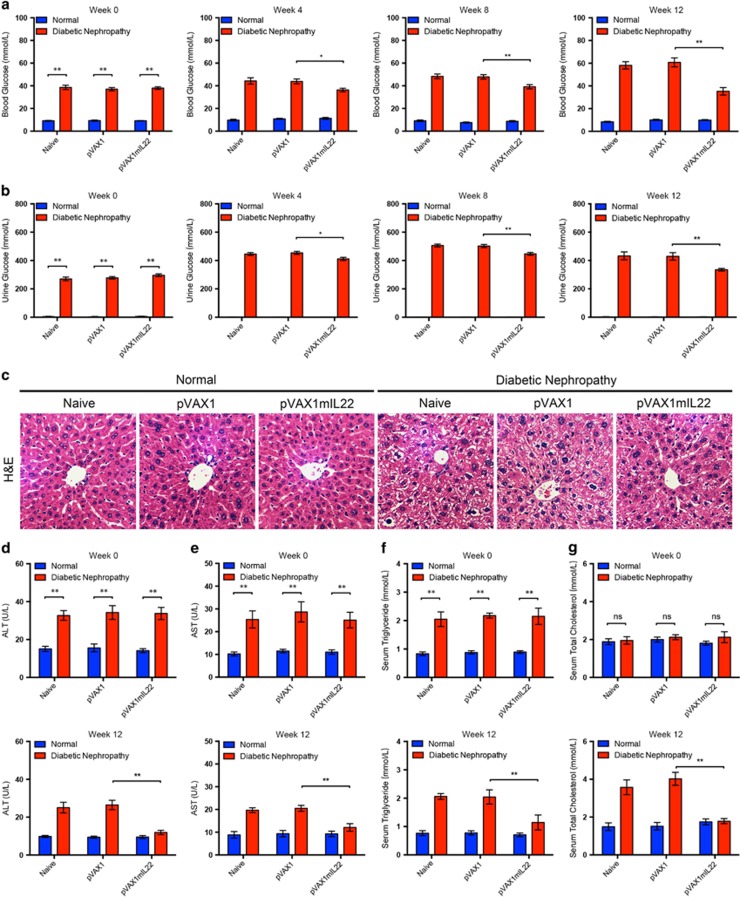
Improved glycemic and metabolic control in diabetic mice after IL-22 gene therapy. (**a**and**b**) Blood glucose levels and urine glucose levels 0, 4, 8, and 12 weeks after IL-22 gene therapy. (**c**) Representative micrographs of H&E staining to detect liver histopathological alterations. Original magnification: × 200. (**d**and **e**) Serum levels of ALT and AST before and after IL-22 gene therapy. (**f** and **g**) Serum levels of triglyceride and total cholesterol before and after IL-22 gene therapy. *N*=5; *P<0.05; **P<0.01. The time point of the initiation of IL-22 gene therapy was defined as week 0. NS, no significance

It has been universally recognized that systemic pathogenesis such as hyperglycemia and metabolic disorders contributes to subsequent renal complications in diabetes. Thus, we explored whether IL-22 could modulate metabolism in mice with DN. Interestingly, IL-22 gene transfer in mice with established nephropathy significantly reduced both blood glucose and urine glucose levels, suggesting better glycemic control in diabetic mice by upregulation of IL-22 (Figures 5a and b). Since liver played a central role in metabolic hemostasis with numerous functions, we subsequently evaluated whether liver injury induced by diabetes was alleviated after IL-22 gene transfer. Diabetes-induced liver injury such as hepatic vacuolization and necrosis was markedly attenuated after intramuscular injection of therapeutic plasmid pVAX1mIL22 for 12 consecutive weeks, as was demonstrated by pathological examination (Figure 5c and Supplementary Figure 5). Meanwhile, hepatomegaly in diabetic mice, as indicated by increased liver index, was markedly reduced by IL-22 gene therapy (Supplementary Figure 6). Congruent with histological analysis, elevated serum levels of ALT and AST were significantly decreased in mice with established nephropathy after IL-22 gene therapy (Figures 5d and e). Furthermore, IL-22 effectively improved metabolic syndrome in diabetic mice as indicated by serum levels of triglyceride and total cholesterol that were comparable to those of normal mice (Figures 5f and g). These data suggested that IL-22 gene therapy improved not only hyperglycemia but also metabolic disorders, which might have protective effects on renal complications in diabetes.

### IL-22 gene therapy suppressed activation of renal NLRP3 inflammasome in mice with established nephropathy

**Figure 6:**
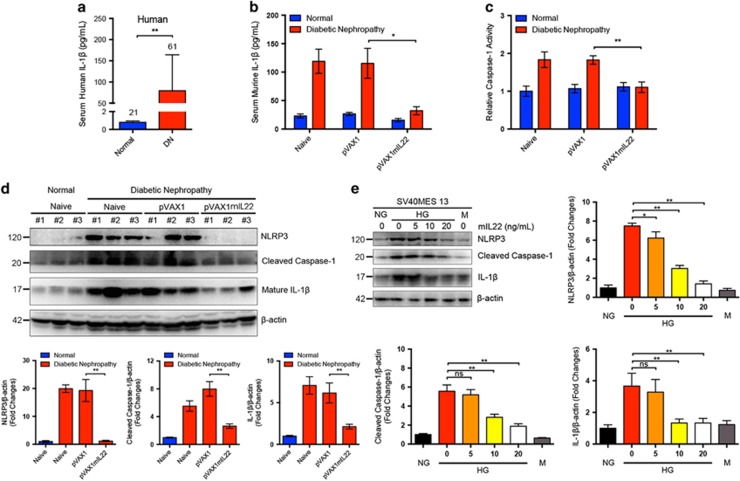
Suppression of renal NLRP3 inflammasome activation by IL-22 gene therapy in mice with established nephropathy. (**a**) Serum levels of human IL-1*β* in patients with DN and age-matched healthy controls measured by ELISA. Numbers of human serum samples analyzed in each group were indicated at the top of each bar. (**b**) Serum levels of murine IL-1*β* in mice with DN and age-matched normal controls measured by ELISA (*N*=5). (**c**) Relative renal cytosolic caspase-1 activity (*N*=5). (**d**) Representative immunoblots and quantitative analysis of renal cytosolic NLRP3, cleaved caspase-1, and IL-1*β* expression from three individual experiments. Densitometric values of immunoreactive bands were normalized to those of *β*-actin and the results were expressed as fold changes. (**e**) Representative immunoblots and quantitative analysis of NLRP3, cleaved caspase-1, and IL-1*β* expression in high glucose-induced renal glomerular mesangial cells from three independent experiments. Densitometric values of immunoreactive bands were normalized to those of *β*-actin and the results were expressed as fold changes. *P<0.05; **P<0.01

Mounting evidence have demonstrated that the NLRP3 inflammasome is an important contributor to inflammation via caspase-1-mediated processing, and secretion of the pro-inflammatory cytokines and activation of NLRP3 inflammasome plays a critical role in the pathogenesis of DN. Thus, we explored whether IL-22 could modulate NLRP3 inflammasome activation in DN. As shown in Figure 6a, serum levels of human IL-1*β* were remarkably elevated in patients with DN as compared with age-matched healthy controls. Meanwhile, serum levels of murine IL-1*β* were also significantly increased in STZ-induced experimental diabetic mice, but drastically decreased after IL-22 gene therapy (Figure 6b). Interestingly, caspase-1 activity assay demonstrated that IL-22 intervention tended to normalize upregulated caspase-1 activity in renal tissues of mice with DKD (Figure 6c). Consistent with previous studies, DN could lead to renal activation of NLRP3 inflammasome, cleavage of caspase-1, and secretion of mature IL-1*β* as indicated by immunoblot analysis. Conversely, IL-22 gene therapy drastically reversed the activation of NLRP3, cleavage of caspase-1, and the maturation of IL-1*β* in renal tissues, suggesting unexpected anti-inflammatory function of IL-22 via suppressing the activation of NLRP3 inflammasome *in vivo* (Figure 6d). In addition, IL-22 markedly downregulated high glucose-induced activation of NLRP3 inflammasome in mouse renal mesangial cells in a dose-dependent manner, indicating that the effects of IL-22 on NLRP3 inflammasome activation was independent of improved glycemic control (Figure 6e). These data indicated that IL-22 gene therapy almost abolished activation of NLRP3 inflammasome, through which IL-22 exerted anti-inflammatory effects in DN. Collectively, we found that IL-22 gene therapy could exert favorable effects on established DN via simultaneously alleviating systemic metabolic syndrome and downregulating renal NLRP3 inflammasome activation (Supplementary Figure 7).

## Discussion

This study focused on deciphering the therapeutic effects and underlying molecular mechanisms of IL-22 for the treatment of DKD. In the current study, on the basis of identifying the possible association between the downregulation of IL-22 and the progression of DN, we provided *in vivo* evidence for the first time that IL-22 significantly alleviated renal injury and fibrosis in established DN. Notably, we reported for the first time that IL-22 gene therapy not only suppressed the systemic pathogenesis of DN, including hyperglycemia and metabolic disorders, but also protected against renal pathogenesis via suppression of NLRP3 inflammasome activation. These findings suggested that IL-22 might have therapeutic potential for the treatment of DN. Regardless of exactly how IL-22 controls metabolic syndrome and regulates NLRP3 inflammasome activation, our results have revealed possible molecular mechanisms responsible for therapeutic effects of IL-22 for DKD and pointed out a comprehensive therapeutic strategy by IL-22 for DN, which is of great significance to the control of diabetic kidney complications.

It has been generally recognized that the characteristic pathological alteration associated with DN is the accumulation of ECM components in glomeruli, often resulting in the development of glomerulosclerosis and loss of renal function. In this study, we found that IL-22 gene therapy could attenuate renal fibrosis through inhibition of ECM accumulation and mesangial matrix expansion in DN, thus largely preserving renal function of diabetic mice. Although the renoprotective effects of IL-22 in acute kidney injury have been investigated in recent years,^21, 22^ studies on the anti-fibrotic effects of IL-22 in renal diseases are still lacking.^36, 37, 38, 39^ Notably, to our knowledge, the findings from this study revealed for the first time that IL-22 could exert anti-fibrotic effects in kidney disease. Nevertheless, it should be pointed out that the effects of IL-22 on different compartments of the kidney, including the epithelium, mesangium interstitium, and endothelium, still need further elucidation.

To date, no remedies are available to prevent the renal complications of diabetes except for therapies that may slow down the progression of DN through intensive control of glycemia and blood pressure. Recently, it has been reported that IL-22 pathway is indispensable for alleviating metabolic disorders and lack of IL-22 signaling contributed to the development of metabolic syndrome.^18, 19, 20^ Consistent with these results, our data demonstrated that IL-22 gene therapy not only strikingly alleviated hyperglycemia, but also markedly reduced serum levels of triglyceride and total cholesterol in STZ-induced diabetic mice, further unveiling the biological function of IL-22 in metabolism regulation. It has been universally recognized that simply targeting systemic hyperglycemia and metabolic disorders is not sufficient to arrest the progression of DN,^8, 40, 41, 42, 43^ indicating that there must be other molecular mechanism responsible for therapeutic effects of IL-22 for DN.

In addition to the tight control of hyperglycemia and metabolic disorders, anti-inflammation has been considered to be a therapeutic approach to minimize the diabetic complications. It has been elucidated by late-breaking studies that activation of NLRP3 inflammasome as a main contributor to inflammation participates in the onset and progression of DN and has been recognized as a promising therapeutic target for the treatment of DN.^44, 45, 46^ In the current study, we demonstrated that IL-22 was able to reduce systemic and renal inflammation in diabetic mice, consistent with previous studies in mouse model of acute kidney injury.^21, 22^ Interestingly, our findings indicated that IL-22 suppressed renal fibrosis via downregulation of NLRP3 inflammasome activation and subsequent caspase-1-mediated processing of pro-IL-1*β* and secretion of the mature cytokines, therefore suppressing renal fibrosis. It is noteworthy that activation of NLRP3 inflammasome occurred at an early stage of DN as demonstrated by previous studies^30^ and IL-22 gene therapy reversed the activation of NLRP3 inflammasome as indicated by this study. Meanwhile, therapeutic effects of IL-22 gene therapy were apparent despite initiation of treatment 17 weeks after persistent hyperglycemia and nephropathy onset, reflecting at least partial disease reversal by targeting renal NLRP3 inflammasome. It has been reported that IL-22 could promote the activity of NLRC4 for sustained production of the IL-1 receptor antagonist IL-1Ra, thereby restraining NLRP3 activity.^17^ However, we have to point out that so far there is no conclusive evidence that revealed the interrelationship between IL-22 signaling pathway and NLRP3/caspase-1/IL-1*β* pathway and our understanding toward the exact role of IL-22 signaling pathway in the progression of renal fibrosis and DN was still far from complete.^36, 47^ Therefore, to identify IL-22 as a novel therapy for DN that simultaneously inhibits the systemic and renal pathogeneses, future studies should explore in-depth the relationship between IL-22 signaling and NLRP3 inflammasome pathway in DKD.

In summary, this study provided new evidence for a better understanding of the biological activities of IL-22 in DN in terms of protection against renal fibrosis and NLRP3 inflammasome activation. Further investigation into multiple biological functions of IL-22, including glycemic control, metabolic regulation, anti-inflammation, and anti-fibrosis, may ultimately help us to identify IL-22 as a novel therapeutic agent for the treatment of DN.

## Materials and methods

### Reagents and antibodies

Streptozocin, d-glucose, and d-mannitol were purchased from Sigma (St Louis, MO, USA). Recombinant human TGF-*β*1 and IL-22 were both from Peprotech (Rocky Hill, NJ, USA) and recombinant mouse IL-22 was from Novoprotein (Shanghai, China). See Supplementary Materials for details of antibodies used for immunoblot and immunohistochemical analysis.

### Patient selection and volunteer recruitment

A total of 61 DN patients (45 males and 16 females) with an average age of 63.31±1.33 years were included in this study. The criteria for inclusion were as follows: diagnosis of type 1 or type 2 DM; disease duration >10 years; and presence of both persistent albuminuria and diabetic retinopathy. The criteria for exclusion were as follows: history of non-diabetic kidney disease; severe hepatic, cardiac or other organ impairment; infection and malignancy. The serum samples of patients with DN were obtained from Changhai Hospital upon informed consent and the study protocol was granted by the Ethics Committee of Changhai Hospital. Serum samples of the non-diabetic control group were obtained from 21 healthy volunteers (14 males and 7 females) with an average age of 63.62±2.42 years and there was no statistical significance in age (*t*=0.115, *P*=0.909) and gender (*χ*^2^=0.391, *P*=0.532) distributions between patients with DN and healthy volunteers. See Supplementary Materials for qualification of serum IL-22 and IL-1*β* in mice and patients.

### Mouse model of experimental DN and *in vivo* intervention study

Male C57BL/6 mice were obtained from the SLAC Laboratory Animal Co. Ltd (Shanghai, China) and maintained under specific-pathogen-free conditions (20~24 °C, 12/12 h light–dark cycle) with free access to food and water. All animal experiments were conducted in accordance with the standards and procedures approved by Animal Ethical Committee of School of Pharmacy at Fudan University. See Supplementary Materials for the establishment of experimental DN and the construction of therapeutic plasmid encoding murine IL-22. Intervention with the recombinant eukaryotic expression plasmid pVAX1mIL22 (100 *μ*g/dose, once a week) was initiated in mice with established DN by bilateral intramuscular injection at 17 weeks after the last STZ injection and continued for 12 weeks to determine the therapeutic effects of IL-22 on the progression of DN. All intramuscular injections were carried out under sodium pentobarbital anesthesia (50 mg/kg body weight, intraperitoneally) to minimize the sufferings of the mice. At the end of the experiment, 6 h urinary samples were collected using metabolic cages and non-fasting serum samples were obtained for the assessment of biochemical parameters.

### Histopathological and immunohistochemical analysis

After the mice were killed under anesthesia, kidney tissues were immediately collected, weighted, cross-sectioned, fixed with 4% formaldehyde, and then processed for histopathological and immunohistochemical analysis. For assessment of kidney injury, renal sections were then stained with H&E for general morphological examination, PAS for glomerulosclerosis evaluation, and Masson for collagen deposition and interstitial expansion assessment, respectively. Mesangial index, presented as the percentage of PAS-positive area in glomerulus, was used to quantify mesangial ECM. Immunohistochemical staining of fibronectin and collagen IV, counterstained with hematoxylin for the nuclei, was performed to clarify the effect of IL-22 gene therapy on renal fibrosis. Positive staining area was quantified using ImageJ software 1.49v (National Institutes of Health, Bethesda, MD, USA) and expressed as a percentage of the total glomerular area. Briefly, 20 glomeruli were randomly selected from each group and positive signals within each glomerulus were highlighted, outlined, and measured by ImageJ. Liver tissues fixed in 4% paraformaldehyde were processed and then stained with H&E for histopathological analysis, as previously described.^48^ Ten fields were randomly selected and the vacuolized hepatic cells were counted manually using the ImageJ plugins cell counter. The percentage of vacuolized hepatic cells was calculated to quantify liver pathological alteration. Histopathological and immunohistochemical analysis were evaluated in a blinded manner by two independent investigators. See Supplementary Materials for biochemical parameters analysis and caspase-1 activity assay.

### Immunoblot analysis

See Supplementary Materials for glomerular mesangial cell and renal tubular epithelial cell culture and treatment. Equivalent amounts of cytosolic proteins extracted from renal tissues or cultured cells were subjected to immunoblot analysis as described previously.^49^ Densitometric values of immunoreactive bands were quantified using ImageJ.

### Statistical analysis

The data were presented as the mean±S.E.M. Statistical analysis was performed with Student’s *t*-test or one-way ANOVA analysis of variance using GraphPad Prism 6.0 Software (GraphPad Software Inc., San Diego, CA, USA). Spearman’s correlation coefficient was used to evaluate the significance of the association between expression of IL-22 and progression of DKD in patients with DN.
