# Supplementary material for: Interleukin-22 ameliorated renal injury and fibrosis in diabetic nephropathy through inhibition of NLRP3 inflammasome activation
Source: Cell Death Dis. 2017 Jul 20;8(7):e2937–. doi: 10.1038/cddis.2017.292 (PMC5550847; doi:10.1038/cddis.2017.292)
Supplement: Supplementary Materials [file cddis2017292x1.pdf]

Interleukin-22 ameliorated renal injury and fibrosis in diabetic nephropathy through inhibition of NLRP3 inflammasome activation

Shaofei Wang[1*], Yubin Li[1*], Jiajun Fan[1], Xuyao Zhang[1], Jingyun Luan[1], Qi Bian[2], Tao Ding[2], Yichen Wang[1], Ziyu Wang[1], Ping Song[1], Daxiang Cui[3], Xiaobin Mei[2], Dianwen Ju[1]

Supplementary Materials and Methods

Antibodies

The primary antibodies used for immunoblot analysis were as follows: anti-IL-22, anti-Fibronectin and anti-Collagen IV (Abcam, Cambridge, Massachusetts, USA); anti-α-Smooth Muscle Actin (α-SMA), anti-Vimentin, anti-Cleaved Caspasse-1, anti-IL-1β and anti-β-actin (Cell Signaling Technology, Danvers, MA, USA); anti-NLRP3 (Epitomics, Burlingame, CA, USA). The secondary antibodies used for immunoblot analysis including peroxidase-conjugated goat anti-rabbit and anti-mouse immunoglobulin G (IgG) were obtained from Jackson ImmunoResearch Laboratory (West Grove, PA, USA). The primary and secondary antibodies for immunohistochemical analysis were obtained from DAKO Corporation (Carpinteria, CA, USA).

Qualification of serum IL-22 and IL-1β in patients and mice

Serum levels of IL-22 in patients and mice were qualified using Human IL-22 Platinum ELISA Kit and Mouse IL-22 Platinum ELISA Kit (eBioscience, San Diego, CA, USA), respectively. Serum levels of IL-1β in patients and mice were determined by ELISA kit (for human samples: MultySciences Biotech, Hangzhou, China; for mouse samples:

Boatman Biotech, Shanghai, China) according to the manufacturer's instructions.

Mouse model of experimental DN

In the current study, we used a long-term model of STZ-induced DN to test therapeutic intervention. Briefly, persistent hyperglycemia was induced in 8-week-old male C57BL/6 mice with a mean body weight of 20g by a daily intraperitoneal injection of STZ (50mg/kg body weight, freshly dissolved in 100 mM sterile sodium citrate buffer, pH 4.5) for 5 consecutive days. Age-matched mice intraperitoneally injected with sodium citrate buffer alone for 5 consecutive days served as controls. Nonfasting blood glucose was monitored weekly for the first 2 weeks after the last STZ injection to determine the development of hyperglycemia. Mice were sacrificed and examined for the establishment of DN by histopathological analysis of PAS-stained renal sections at 16 weeks after the last STZ injection.

Construction of therapeutic plasmid DNA encoding murine IL-22

Nucleotide sequence encoding murine IL-22, synthesized by GeneScript Biotech (Nanjing, China), was sub-cloned into the *Kpn I* and *BamH I* sites in the pVAX1 therapeutic plasmid vector (Invitrogen, Carlsbad, CA, USA). The resulting plasmids pVAX1mIL22 were transformed into *E.coli* competent cells Top10 (TIANGEN Biotech, Beijing, China) and verified by sequencing analysis. Subsequently, the recombinant pVAX1mIL22 plasmids and empty vector pVAX1 plasmids were prepared in large amounts using the EndoFree Plasmid Maxiprep Kit (Biotool, Houston, TX, USA). The concentration and purity of endotoxin-free plasmids were measured by Nanodrop 2000 Spectrophotometer (Thermo Scientific, Waltham, MA, USA).

Expression of IL-22 *in vitro*

Human embryonic kidney cells HEK293T, maintained in DMEM medium supplemented with 10% fetal bovine serum (FBS), penicillin (100 U/mL) and streptomycin (100 μg/mL), were obtained from Cell Bank of Shanghai Institutes for Biological Sciences, Chinese Academy of Sciences (Shanghai, China). The endotoxin-free pVAX1mIL22 or pVAX1 plasmids were transfected into HEK293T cells using DNA Transfection Reagent (Biotool, Houston, TX, USA) according to the manufacturer's instructions. The transfected cells were harvested at 24 h post transfection and the expression of IL-22 in transfected cells was confirmed by immunoblot analysis.

Expression of IL-22 *in vivo*

To determine the *in vivo* expression of murine IL-22, 8-week-old male C57BL/6 mice were randomly divided into three groups (five mice in each group): (1) naive control group (2) pVAX1 empty vector control group (3) pVAX1mIL22 therapeutic intervention group. 100 μg of the recombinant plasmid pVAX1mIL22 or empty vector pVAX1 were administered to mice by bilateral intramuscular injection under sodium pentobarbital anesthesia. Serum levels of IL-22 at indicated time points were determined by ELISA according to the manufacturer's instructions.

Biochemical parameters analysis

Serum creatinine, blood urea nitrogen (BUN) and proteinuria were determined to evaluate kidney function. Serum levels of alanine aminotransferase (ALT) and aspartate aminotransferase (AST) were measured to assess liver injury. The related detection kits

for biochemical parameters analysis were all purchased from Nanjing Jiancheng Bioengineering Institute (Nanjing, China) and used according to the manufacturer's instructions.

Caspase-1 activity assay

The enzymatic activity of Caspase-1 in renal tissue was determined by Caspase-1 Activity Detection Kit (Beyotime Institute of Biotechnology, Beijing, China) according to the manufacturer's protocol on the basis of ability of Caspase-1 to change acetyl-Tyr-Val-Ala-Asp p-nitroanilide (Ac-YVAD-pNA) into the yellow formazan product p-nitroaniline (pNA).

Glomerular mesangial cell and renal tubular epithelial cell culture and treatment

Mouse glomerular mesangial cells SV40MES13 and human renal tubular epithelial cells HK-2 were obtained from Cell Bank of Shanghai Institutes for Biological Sciences, Chinese Academy of Sciences (Shanghai, China) and maintained in RPMI1640 medium supplemented with 10% fetal bovine serum (FBS), penicillin (100 U/mL) and streptomycin (100 μg/mL) in a 95% air and 5% CO2 atmosphere at 37 ℃. To explore the effect of IL-22 on high glucose-induced ECM synthesis, cells were then incubated in medium containing NG (normal glucose, 5 mM D-glucose), HG (high glucose, 30 mM D-glucose), or M (5 mM D-glucose + 25 mM D-mannitol; osmotic control) with or without murine IL-22 co-treatment for 24h as indicated. To explore the effect of IL-22 on TGF-β1-induced ECM synthesis, cells were stimulated in medium with or without TGF-β1 (10ng/mL) in the absence or presence of IL-22 for 24h as indicated. And then cells were harvested, lysed and processed for western blot analysis.

Supplementary Figures

Supplementary Fig. 1

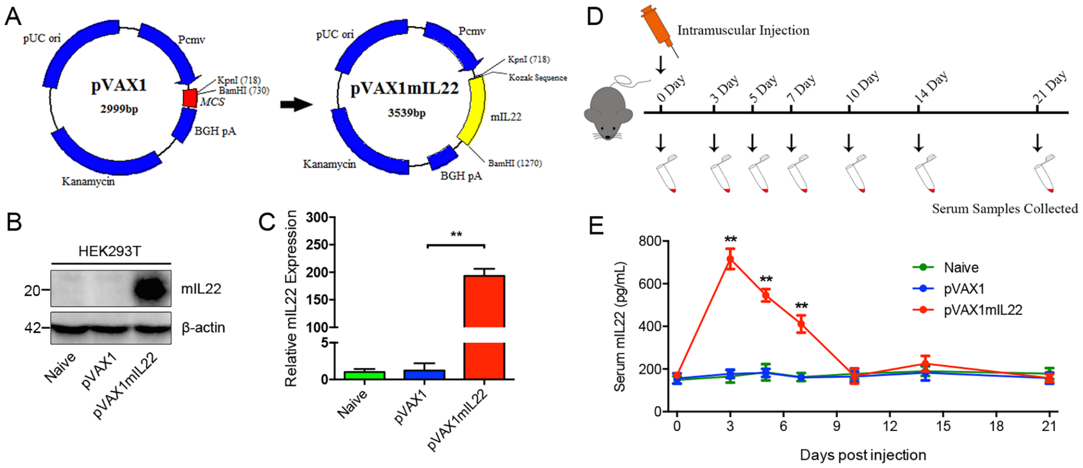

Supplementary Fig. 1 Construction of therapeutic plasmid DNA encoding IL-22 and expression of the target protein *in vitro* and *in vivo*.

(A) Schematic diagram showing the construction of recombinant eukaryotic expression plasmid pVAX1mIL22. (B) Expression of IL-22 in transiently transfected HEK293T cells 24 h after transfection by immunoblot analysis. (C) Semi-quantification of IL-22 expression in transiently transfected HEK293T cells 24 h post-transfection normalized to β-actin of 3 independent experiments by ImageJ. (D) Scheme of intramuscular injection of recombinant plasmid pVAX1mIL22 and collection of serum samples. (E) Serum levels of IL-22 0, 3, 5, 7, 10, 14, and 21 days after intramuscular injection analyzed by ELISA (N≥3). $^{**}p < 0.01$.

Supplementary Fig. 2

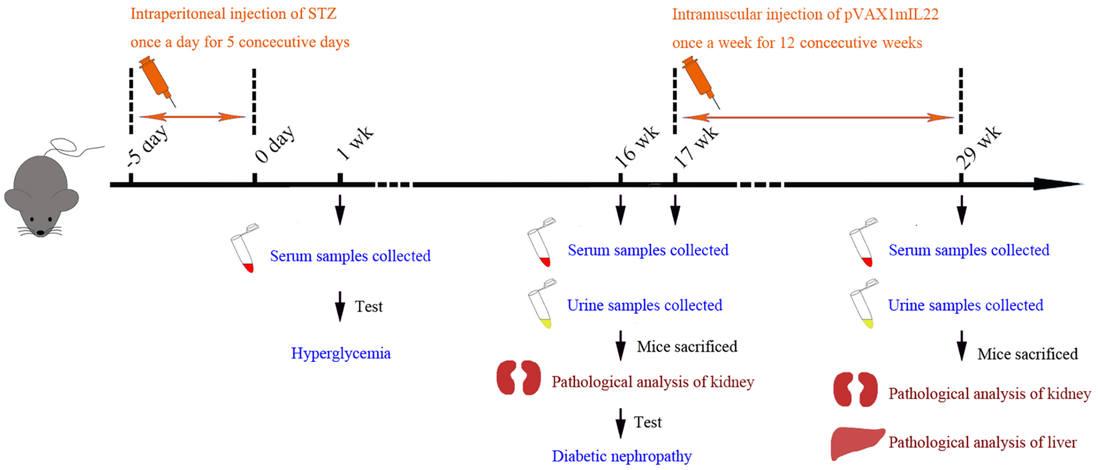

Supplementary Fig. 2 Schematic diagram of establishment of STZ-induced diabetic nephropathy and subsequent therapeutic intervention. IL-22 gene therapy was initiated in mice with established nephropathy at 17 weeks after the last STZ injection and continued for 12 weeks to determine the therapeutic effects of IL-22 on the progression of diabetic nephropathy.

Supplementary Fig.3

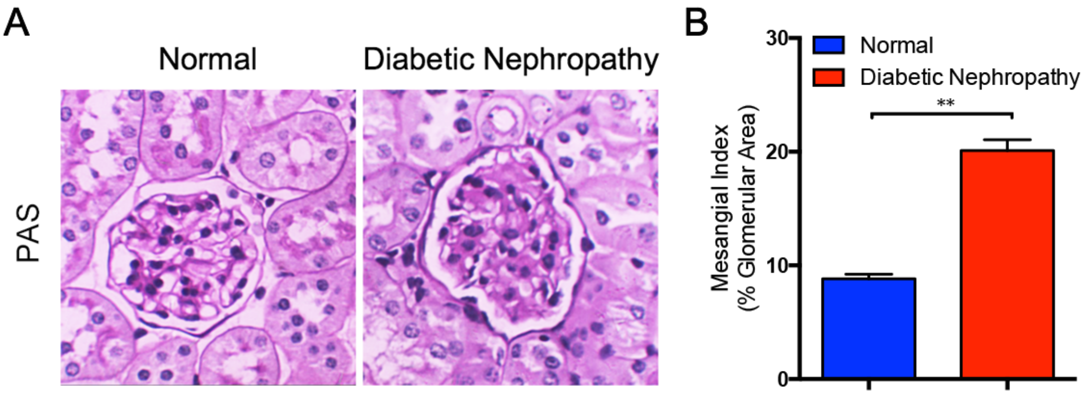

Supplementary Fig. 3 Establishment of diabetic nephropathy as determined by renal histopathological analysis. Mice were sacrificed and examined for the establishment of DKD by histopathological analysis of PAS-stained renal sections at 16 weeks after the last STZ injection. (A) Representative micrographs of PAS staining.

Original magnification: ×400. (B) Glomerular mesangial matrix expansion quantified from PAS staining. **p <0.01.

Supplementary Fig. 4

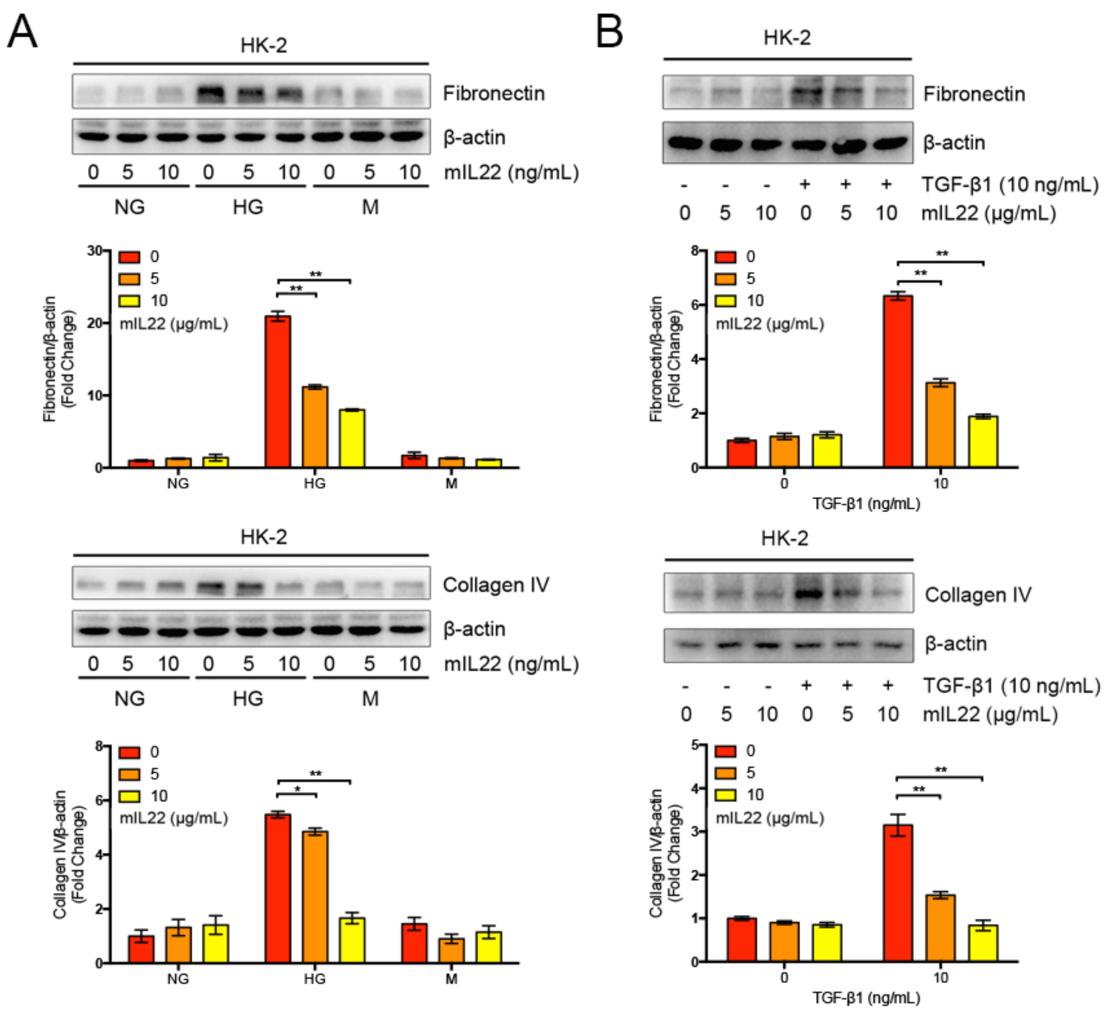

Supplementary Fig. 4 Reduced synthesis of high glucose-induced and TGF-β1-induced ECM proteins in human renal tubular epithelial cells after IL-22 treatment. (A-B) Representative immunoblots and semi-quantitative analysis of cytosolic expression of Fibronectin and Collagen IV from 3 independent experiments. Densitometric values of immunoreactive bands were normalized to those of β-actin and the results were expressed as fold changes. ns, no significance; *p <0.05; **p <0.01.

Supplementary Fig. 5

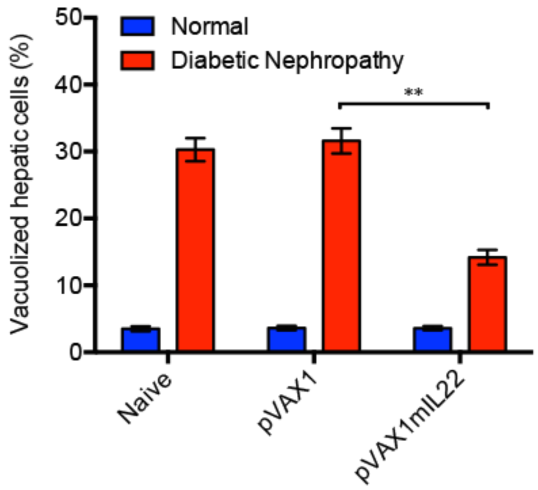

Supplementary Fig. 5 Quantification of liver pathological alteration in diabetic mice after IL-22 gene therapy. The results were expressed as the percentage of vacuolized hepatic cells. $**p < 0.01$.

Supplementary Fig. 6

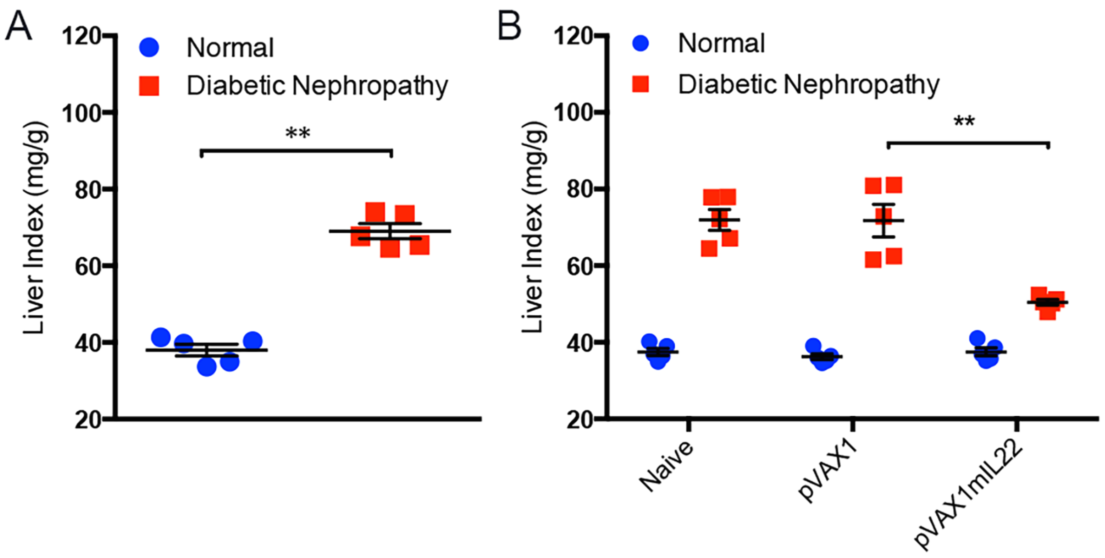

Supplementary Fig. 6 Reduced Liver index of mice with established nephropathy after IL-22 gene therapy. Liver index of mice with established nephropathy (A) before and (B) after IL-22 gene therapy. Liver index=Liver weight (mg)/Body weight (g). N=5; $**p < 0.01$.

Supplementary Fig. 7

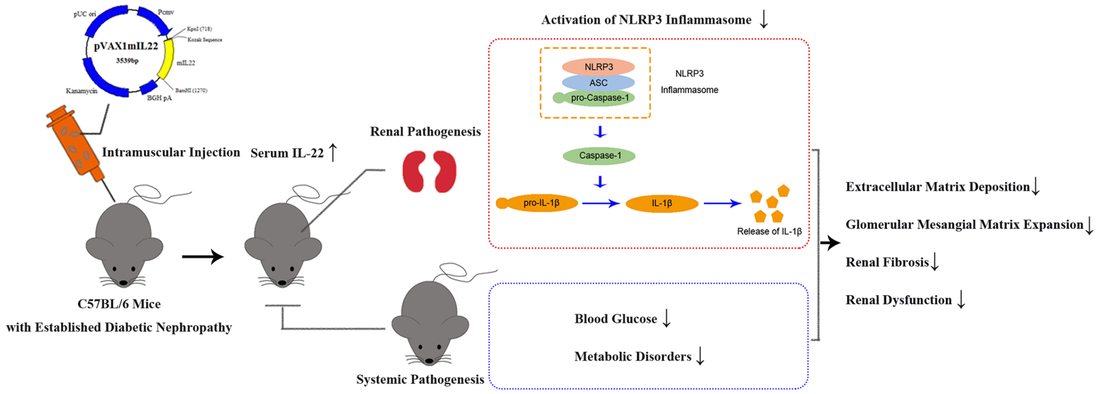

Supplementary Fig. 7 Schematic diagram showing the potential therapeutic effects and underlying mechanisms of IL-22 in the treatment of diabetic nephropathy. IL-22 gene therapy could exert favorable effects on diabetic nephropathy via simultaneously alleviating systemic metabolic syndrome and downregulating renal NLRP3/Caspase-1/IL-1β pathway, consequently attenuating ECM deposition and subsequent renal fibrosis.
